# Experiences of a sensitization program on common mental disorders for primary care physicians using problem-based learning approach

**DOI:** 10.4103/0019-5545.58296

**Published:** 2009

**Authors:** Rakesh K. Chadda, Mamta Sood, Nand Kumar

**Affiliations:** Department of Psychiatry, All India Institute of Medical Sciences, Ansari Nagar, New Delhi - 110 029, India

**Keywords:** Common mental disorders, primary care, sensitization program

## Abstract

Authors describe their experience of conducting a series of one-day sensitization programs on common mental disorders for primary care doctors. One hundred and two doctors attended the program. A problem-based learning approach involving active participation by the participants was used. The program was conducted as a part of the educational activities under the National Mental Health Program of India. It is suggested that this approach can be used effectively to impart training to mental health professionals in low-income countries like India.

## INTRODUCTION

India with its population of more than one billion has nearly 100 million people suffering from mental and neurological problems, who require professional help at any point in time.[1] The country's budget allocation to health is only 5.2% of the GDP, with mental health forming a meager 0.83% of the total health budget.[2] The mental health resources are very low, compared to high-income countries, comprising just 0.25 psychiatric beds per 10,000 population, 0.2 psychiatrists, 0.03 clinical psychologists, 0.05 psychiatric nurses, and 0.03 social workers per 100,000 of the population.[3] Thus, there is a gross disparity between resources and needs. The country trains 230 psychiatrists every year,[4] but many of them relocate to high-income countries for greener pastures.[3]

Considering the limited resources available, the Government of India had launched its National Mental Health Program (NMHP),[5] in 1982, with the objectives of ensuring availability and accessibility of minimum mental health care for all, integration of mental health into general health care and promotion of community participation. The original program was very ambitious with a vision of having a psychiatrist in every district hospital besides training the primary care doctors and health workers. The approach was modified in the mid 1990s by adopting selected districts in the country and taking a district as a unit for the program. Activities under the program included training of the medical officers, paramedical staff, and the community leaders in mental health, provision of community outreach clinics and a central specialty unit at the district hospital. Till recently, about 100 districts in the country have been covered.[6]

## TRAINING OF PRIMARY CARE DOCTORS IN INDIA UNDER THE NATIONAL MENTAL HEALTH PROGRAM

Under the National Mental Health Program, the medical officers working in the district are provided a two-week refresher course in psychiatry in batches. The course consists of mainly theoretical lectures covering various psychiatric illnesses and some case demonstrations. There has not been much follow up of the doctors who had been trained, on how the program had benefited them, although definitely it had been able to sensitize them to mental health issues. Similarly, there is a provision of shorter sensitization programs, lasting a few days, for health workers and community leaders.

Medical officers who work in primary health care centers are the first contact for any health-related problems. In their routine clinical practice, they are unlikely to see patients with severe mental illnesses, but more often see patients with common mental disorders like depression, anxiety disorders, and somatoform disorders. The prevalence of common mental disorders in general/primary health care settings has been reported to vary from 20-45%.[7] Such illnesses, if left untreated, can cause significant functional impairment. Thus, it is important for the primary care physicians to be familiar with these disorders so as to identify and manage such patients effectively in their clinical practice.

Directorates of Health Services of different states of the country, in their independent capacity, also look after various national health programs. The authors were requested by the Director Health Services, Government of Delhi (India), to conduct a series of one-day sensitization programs on common mental disorders for the medical officers, working in various primary care clinics in the state of Delhi. The program was kept for a duration of just one day because of logistic issues, as deputing medical officers from their respective clinics for long periods often jeopardized the general health services. Earlier, it had been reported that after a two weeks' short-term training in mental health care, medical officers demonstrated a significant gain in knowledge.[8,9] However, it is often difficult for doctors working in primary care settings to be sent for two weeks training.

The learning objectives of the workshop were so that the medical officers would be able to identify and diagnose these disorders in patients presenting in their day-to-day care; to decide the basic investigations required for ruling out the underlying organic cause or physical comorbidity, and decide the management, both pharmacological treatment as well as minimal skills for nonpharmacological interventions, including reassurance, encouragement, and psychoeducation of the patient and the family.

## DESCRIPTION OF PROGRAM

The program was conducted by a team of three psychiatrists from the Department of Psychiatry, All India Institute of Medical Sciences, New Delhi (India). At the beginning of the program, an overview was presented, which highlighted the aims and objectives of training and also the structure of the program. The program consisted of three sessions, each session devoted to anxiety disorders, depressive disorders, and somatoform disorders, respectively. These conditions were chosen because these are common and remain undetected, as these are not as obvious as the serious mental disorders like schizophrenia, are associated with significant dysfunction and medical comorbidity, and are easy to treat in primary care settings. It was intended to have a group of about 40 doctors, which could be divided into five subgroups of eight each. A total of three such workshops were conducted and 102 medical officers attended the program.

To ensure active involvement of the participants in the present workshop, a problem-based learning approach was used.[10] Problem-based learning (PBL) is a small group teaching method; working on defined problem areas to increase knowledge and understanding. Presentation of clinical material as a stimulus for learning enables the participants to understand the relevance of the underlying scientific knowledge and its application in clinical practice. Besides acquisition of knowledge, learning in a group facilitates communication skills, teamwork, finding solutions to the problems, taking independent responsibility for learning, sharing information, and respect for others. In the present program, paper-based PBL scenarios were chosen, to ensure that all the participants were exposed to the same problems.

The participants were divided into groups of six to eight each. Each session comprised of a group task, presentation of the problem solution by one of the group members and a brief discussion. Each group was given a problem in the form of case vignette along with five questions about the case, such as, further information required to make a diagnosis, provisional diagnosis, differential diagnosis, and treatment. They were also provided with a pen and paper to prepare their presentation for an overhead projector. The case vignettes represented various anxiety disorders, depressive disorders, and somatoform disorders. These open-ended questions were chosen to generate discussion in the group. The groups were given 10 minutes for the task followed by 5 minutes for presentation and discussion. This was followed by an interactive presentation on the topic by one of the psychiatrists, followed by a general discussion.

## DISCUSSION

In India, in most of the training programs for medical officers in the past, a lecture format has been followed by a question-answer session. A visit to an outpatient clinic or a psychiatric ward was also a part of the program. In such programs, the participants were more often the passive recipients, although they were given an opportunity to ask questions. Such programs offered limited opportunity for active participation from the trainees, and were associated with low receptivity by the learners.

It was hoped that PBL approach would allow flexibility, exchange of ideas, and create an atmosphere where all the participants would be free to express their opinion. Varied experiences of individual group members were incorporated to enrich the discussion. There would be more scope for giving and taking of information, opinions, suggestions, and clarifications.

At the end of the presentation, a 10 minute period was given for getting feedback from administrators and participants. The program was well received and it was observed that most participants were active in the discussion. The program format was perceived to be very useful by most, as it had made them participate in the learning, and they had an opportunity to voice the day-to-day practical difficulties they faced while dealing with such cases during their clinical practice, and the solutions offered were easy to implement at the primary health care level.

## CONCLUSION

The problem-based learning approach, as reported in this article, may be used in various continuing medical education programs for medical officers and paramedical professionals. By using such an approach, it is possible to tailor the training according to the needs of the group to be trained. Such a model offers a better scope for learning and has utility for their future clinical practice.
